# Magnetic resonance angiography: current status and future directions

**DOI:** 10.1186/1532-429X-13-19

**Published:** 2011-03-09

**Authors:** Michael P Hartung, Thomas M Grist, Christopher J François

**Affiliations:** 1 Department of Radiology, School of Medicine and Public Health, University of Wisconsin-Madison, Madison, WI

## Abstract

With recent improvement in hardware and software techniques, magnetic resonance angiography (MRA) has undergone significant changes in technique and approach. The advent of 3.0 T magnets has allowed reduction in exogenous contrast dose without compromising overall image quality. The use of novel intravascular contrast agents substantially increases the image windows and decreases contrast dose. Additionally, the lower risk and cost in non-contrast enhanced (NCE) MRA has sparked renewed interest in these methods. This article discusses the current state of both contrast-enhanced (CE) and NCE-MRA. New CE-MRA methods take advantage of dose reduction at 3.0 T, novel contrast agents, and parallel imaging methods. The risks of gadolinium-based contrast media, and the NCE-MRA methods of time-of-flight, steady-state free precession, and phase contrast are discussed.

## Introduction

Clinical applications for Magnetic Resonance Angiography (MRA) are rapidly expanding as technological advances in both hardware and imaging techniques overcome previous limitations, and the risks from intravenous contrast agents and repeated ionizing radiation exposure become more salient for the clinician and patient [1]. Magnetic resonance imaging (MRI) has the advantage of relying on the intrinsic magnetic properties of body tissues and blood in an external magnetic field to produce an image, without the need of ionizing radiation or nephrotoxic contrast agents. With the increasing availability and use of 3.0-Tesla (T) magnets, which received FDA approval in 2002, and optimized pulse sequences, high-quality images with excellent spatial resolution can be obtained in shorter scan times with smaller or no injections of contrast agents. In this manuscript we will review recent developments in (1) performing MRA at 3.0T, including "low-dose" contrast-enhanced (CE) MRA, and (2) new non-CE (NCE) MRA techniques.

### MRA at 3.0T

**Figure 1:**
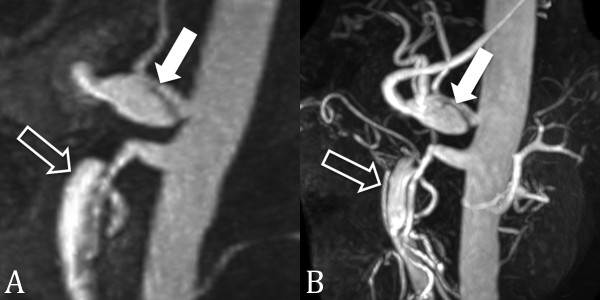
**CE MRA at 1.5 T and 3.0 T**. 56 year-old male with celiac (closed arrow) and superior mesenteric artery (open arrow) dissections. CE MRA at 1.5 T (A) has lower spatial resolution and contrast-to-noise-ratio than at 3.0 T (B).

At 3.0T, twice as many protons are aligned with the magnetic field as compared to 1.5T, resulting in a theoretically doubled signal-to-noise ratio (SNR). This gain in SNR can be taken advantage of to increase the spatial resolution, decrease acquisition time, or a combination of the two to achieve the same SNR characteristics as 1.5T in less time. Increased spatial resolution allows for improved visibility of lesions, and faster acquisition times helps reduce motion artifact and decrease breath-holding requirements [2]. Additionally, the vessel to background contrast enhancemnt effects of gadolinium (Gd) are even more pronounced at 3.0T, producing higher contrast images and therefore requiring lower doses of Gd-based agents to achieve similar image quality found at lower field strengths (Figure 1) [3].

Typically, CE-MRA techniques are used more often than NCE-MRA techniques. Advantages of CE-MRA relative to other MRA techniques, such as time-of-flight (TOF) and phase-contrast (PC), include shorter acquisition times, improved anatomical coverage, and decreased susceptibility to artifacts caused by blood flow and pulsatility. To avoid combined arterial and venous enhancement, shorter acquisition times are necessary to obtain purely "arterial" phase images. This can be done using acquisitions with a parallel imaging or time-resolved techniques. At 3.0T, the gain in SNR can allow higher acceleration factors in parallel imaging to decrease scan times and improve spatial resolution even further [3-5].

While 3.0T opens many possibilities for the future of MRA, it also carries with it a new set of clinical and technological problems that need to be addressed before gaining widespread use. Pulse sequences that have been optimized for 1.5T may need to be adjusted for 3.0T applications. Additionally, the high magnetic field strength increases energy deposition in the patient and field inhomogeneity, as discussed below.

#### Contrast-enhanced MRA at 3.0T

**Figure 2:**
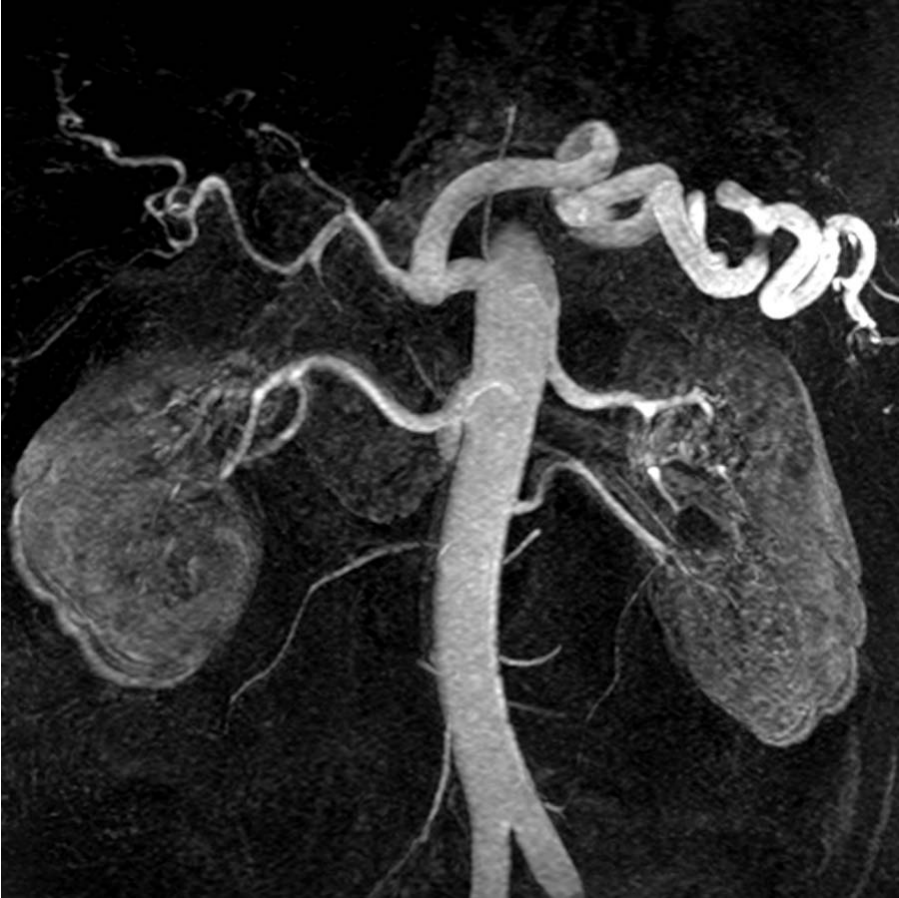
**Low dose CE MRA**. Contrast-enhanced renal MRA at 3.0T using 0.1 mmol/kg of gadobenate dimeglumine. Image quality and vessel conspicuity are excellent even with a relatively low dose of intravenous contrast.

Although gadolinium based agents have an excellent safety record, reports linking gadolinium to nephrogenic systemic fibrosis have sparked renewed interest in "low-dose" CE-MRA and NCE-MRA [6-8]. In addition, low-doses of contrast also help reduce the costs of performing CE-MRA. Gadolinium chelates are paramagnetic compounds that shorten T1 and T2 relaxation times by disrupting spin-lattice and spin-spin interactions respectively. These effects of Gd on body tissues are relatively unaffected by increased magnetic field strength. Thus, although body tissue T1 relaxation times are increased at 3.0T, the T1 relaxation times of Gd-contrast agents remain relatively unchanged at higher magnetic field strengths. This results in noticeable increases in the blood pool-to-background contrast-to-noise ratio (CNR) compared to 1.5T. The increase in CNR at 3.0T can be used to improve the image quality using the same amount of contrast or to decrease the amount of IV contrast injected compared to a similar scan at 1.5T (Figure 2) [5,9]. Tomasian et al. recently demonstrated that for 3.0T MRA of the supraaortic arteries, a contrast dose reduction from 0.15 to 0.05 mmol/kg did not compromise image quality, acquisition speed, or spatial resolution [5]. Arterial occlusive disease was detected nearly equally between the two readers, with no significant difference in arterial definition scores.

CE-MRA has been established as a non-invasive alternative to conventional angiography in evaluating peripheral vascular disease [10-12] and can be an alternative to CTA for the diagnosis of acute pulmonary embolism [13]. Lower-extremity MRA is typically associated with the highest contrast dose protocols of all MR imaging techniques, often requiring a double-dose (0.2 mmol/kg) or more of Gd-contrast to be administered [14]. It has been shown that the amount of Gd-contrast needed at 3.0T for lower extremity MRA can be reduced up to one-third of that used at 1.5T (i.e. from 0.3 mmol/kg to 0.1 mmol/kg) [15]. The resulting images at lower contrast doses had better arterial definition than high-dose images, presumably due to lower residual background signal from the initial contrast injection and less venous contamination [16].

Renal CE-MRA quality at 3.0T has also been evaluated with low-dose Gd. Attenberger et al. demonstrated equal image quality for evaluation of the renal arteries comparing 0.1 mmol/kg of gadobenate dimeglumine at 3.0T with 0.2 mmol/kg of gadobutrol at 1.5T [17]. Kramer et al. compared low-dose (0.1 mmol/kg) gadopentetate dimeglumine at 3.0T with conventional digital subtraction angiography (DSA) for evaluation of renal artery stenosis in 29 patients, yielding good to excellent quality images with sensitivity and specificity of 94% and 96% respectively [4]. These findings suggest that at 3.0T, the contrast dose in current practice is likely higher than needed, and can be lowered without negatively impacting spatial resolution or overall image quality.

**Figure 3:**
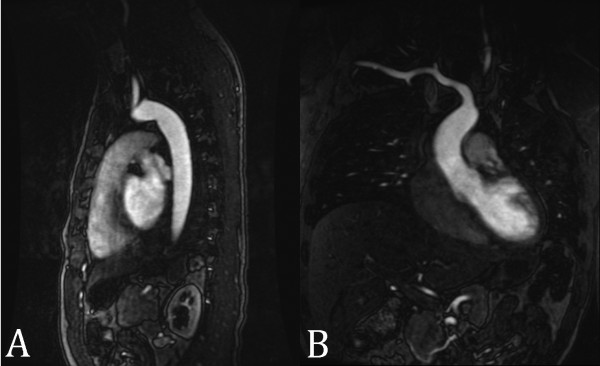
**CE MRA with intravascular contrast agent**. (A) First-pass and (B) steady-state multiplanar reformatted images from contrast-enhanced MRA done with 0.03 mmol/kg of gadofosveset trisodium in a 25 year-old male with a right lower lobe segmental pulmonary embolus (arrow). Even during the steady-state there is substantial intravascular signal to accurately diagnose the pulmonary embolism.

Current CE-MRA techniques using conventional Gd-contrast agents are limited by the need to acquire images relatively quickly during the first pass of contrast material through the vessels of interest. Newer, intravascular Gd-based contrast agents can help overcome these limitations. Gadofosveset trisodium, a protein-binding intravascular contrast agent that has recently obtained FDA approval for use in CE-MRA of the aorto-iliac segments, differs from other gadolinium-based contrast media by having a considerably longer intravascular lifetime and higher relaxivity [18]. Gadofosveset requires smaller total amounts of contrast (Figure 3) and extends the imaging windows up to 60 minutes or more. Images can then be obtained during the steady-state phase after the administration of IV contrast, permitting longer scan times to acquire very high spatial resolution CE-MRA images. A study by Klessen et al. [18] demonstrated that 10 mL of Gadofosveset trisodium produced qualitatively better images with higher arterial contrast compared to 30 mL of gadopentetate dimeglumine. Further optimization of the injection protocol is speculated to further improve the results found in this study.

#### Parallel imaging at 3.0T

**Figure 4:**
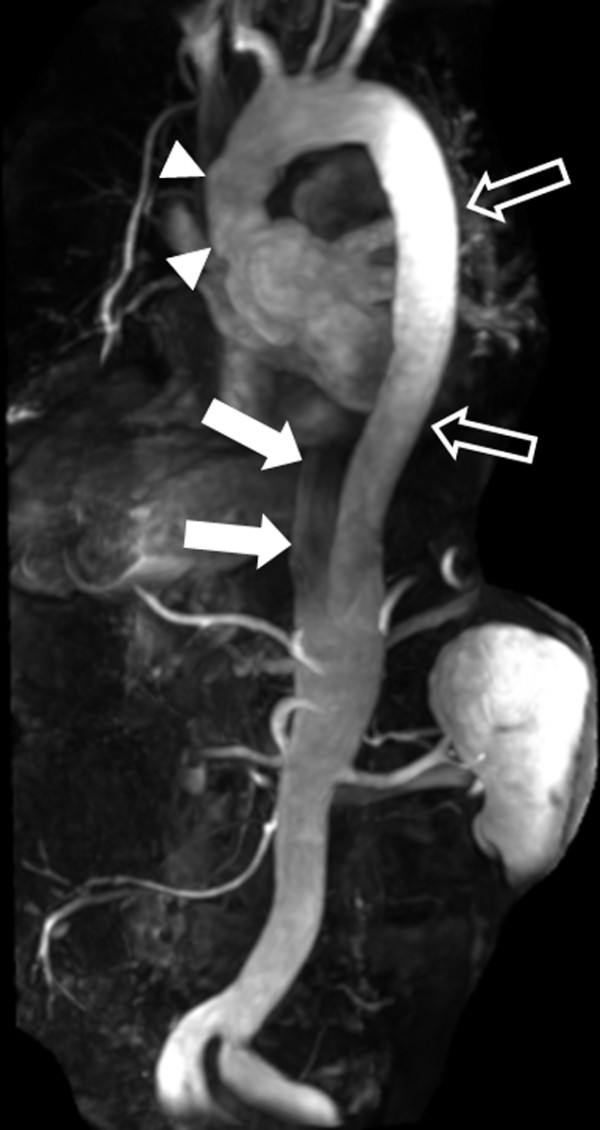
**Large field of view CE MRA using parallel imaging**. Parallel imaging and a 32-channel coil were used to scan the entire aorta from the aortic root to beyond the bifurcation in this 49 year-old male with prior ascending aortic dissection repair (arrowheads) and residual dissection in the descending aorta (open arrows = true lumen; closed arrows = partially thrombosed false lumen).

**Figure 5:**
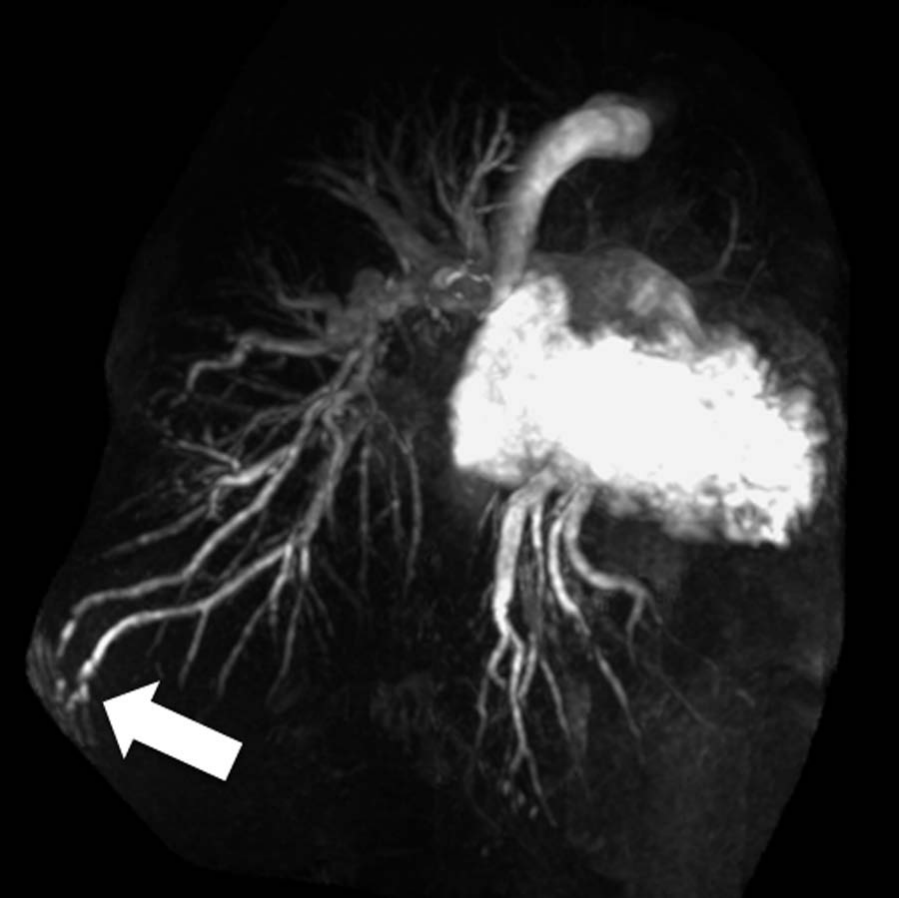
**Rapid whole chest CE MRA using parallel imaging**. Contrast-enhanced pulmonary MRA in 47 year-old male with pulmonary artery hypertension and a pulmonary arteriovenous malformation (arrow). The use of two-dimensional parallel imaging enables the scan time to be reduced to 16 seconds while maintaining whole chest coverage. Imaging at 3.0T increases the contrast-to-noise ratio, even when only using 15 mL of gadobenate dimeglumine as in this case.

**Figure 6:**
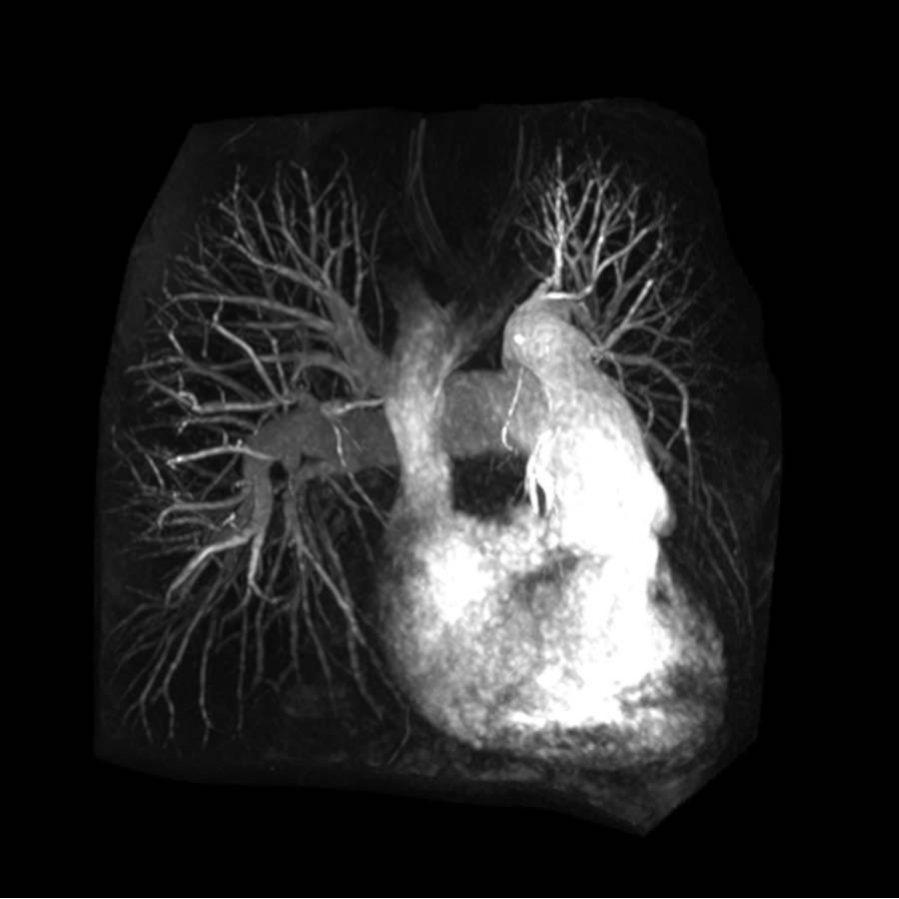
**Rapid whole chest CE MRA using parallel imaging**. The use of parallel imaging to reduce scan time is particularly important in patients who have difficulty holding their breath. This contrast-enhanced pulmonary MRA is from a 42 year-old female with primary pulmonary artery hypertension who requires the use of oxygen. In this case the scan time was 16 seconds.

Parallel imaging further enhances the benefits of 3.0T by undersampling the area of interest as a tradeoff for increased image acquisition speed. Parallel imaging has been applied to CE-MRA to reduce scan time and improve spatial resolution by improving anatomic coverage and removing aliasing artifact with the use of multiple channel coils (Figure 4) [19]. The individual coils, which have varying spatial sensitivities, are used to simultaneously receive MR signal following a single radiofrequency (RF) pulse. This permits faster image acquisition with fewer motion artifacts, less RF excitations pulses, and lower energy burden for the patient, but some aliasing is present due to missing k-space data as a result of undersampling. In a study by Fenchel et al. [20], high-quality CE-MRA with integrated parallel acquisition technique (iPAT2) and single contrast injection has been shown to produce adequate image quality of the entire arterial vasculature with acceptable SNR and CNR values for whole-body applications, in less than 60 seconds. Parallel imaging can also increase the anatomical coverage. Lum, et al. [21] recently demonstrated the use of a two-dimensional autocalibrating parallel imaging technique (2D-ARC) to increase the coverage for abdominal CE-MRA. Subjective image quality and vessel conspicuity were graded for healthy volunteers and patients with suspected renovascular disease for MRA with and without 2D-ARC. The results demonstrated equivalent image quality in both methods, with the benefit of a 3.5-fold increase in imaging volume and complete abdominal coverage within the same acquisition time for 2D-ARC MRA. This same technique can also be used to perform high resolution, whole chest MRA in a shorter time, which is important in the evaluation of patients suspected of having pulmonary embolism or who are short of breath (Figures 5, 6).

#### Limitations and safety concerns for CE-MRA at 3.0T

The stronger magnetic field at 3.0T results in significant challenges and limitations that are yet to be fully overcome. Constructive and destructive interference due to RF field inhomogeneity and increased Specific Absorption Rate (SAR) are major concerns when imaging at 3.0T.

RF field inhomogeneity can result in areas of interference and loss of complete anatomic coverage within the image field. At 3.0T, the resonance frequency of protons in water is 128 MHz, double the value in a 1.5T system, which means that the radiofrequency wavelength is halved from 52 cm to 26 cm. This shortened wavelength can span the dimensions of the field of view for abdominal and pelvic imaging, occurring more frequently in persons with a large body habitus [22]. As two RF waves overlap in the imaging field, constructive or destructive interference can result in areas of brightening or darkening respectively. A similar artifact can occur in persons with large amount of fluid in their abdomen (eg. ascites or pregnancy). Electrical current circulates within the fluid under the strong magnetic field and interferes with the RF field pulses resulting in interference [23]. Advances in coil design, such as multicoil transmit body coils, can suppress eddy currents and improve RF field homogeniety at higher field strengths [24]. In addition to improved coil design, new pulse sequences such as three-dimensional tailored RF pulses have been shown to improve homogeneity of the radiofrequency excitation[25].

RF pulses transfer energy to protons within the patient and ultimately generate heat as a byproduct of energy release. Heat produced within the patient can have detrimental physiologic effects and is carefully monitored within the imaging setting, with current limits of total body heating set by the FDA at 4 W/kg for the whole body over a 15 minute period [26,27]. SAR provides an estimate for the energy deposited in the tissue by the RF pulse and increases with the square of the resonance frequency. At 3.0T, the resonance frequency is double that of a 1.5T system, and thus the SAR is increased fourfold [2]. Modified pulse sequences, acquisition techniques, and hardware designs are being developed to aid in management of the increased SAR at higher fields. The use of parallel imaging also provides an important solution to this problem, as the multiple detector coils used to simultaneously encode a larger anatomic region serve to both decrease acquisition time and decrease the number of RF pulses needed to acquire an image.

### Non Contrast-Enhanced Magnetic Resonance Angiography (NCE-MRA)

The widespread use NCE-MRA has been limited by prolonged acquisition times and motion artifacts that favor CE-MRA. However, several factors have contributed to a renewed interest in NCE-MRA methods, including improvements in MR hardware and software and concerns over the safety of gadolinium-based contrast in high-risk patient groups. The latter is particularly concerning, as patients with moderate to severe renal insufficiency and vascular or metabolic disorders are at risk for developing the debilitating and possibly life-threatening disease of nephrogenic systemic fibrosis (NSF) [6-8]. A recent meta-analysis by Agarwal et al. [28] identified the odds of developing NSF were 27 times greater in patients with chronic kidney disease (N = 79/1393, 5.7%) exposed to gadolinium compared to control subjects with chronic kidney disease (N = 3/2953, 0.1%) who did not receive gadolinium. This poses a significant imaging challenge as metabolic syndrome, diabetes and renal disease continue to afflict a larger percentage of the population each year [29]. Also, situations may occur where NCE-MRA is preferred due to difficult IV access or contraindication of IV contrast material. High-resolution CE-MRA usually requires a large bore IV catheter that may be difficult to place in patients who are obese or with poor veins, and IV contrast agents are usually not given during in pregnancy due to teratogenic effects observed in animal studies.

NCE-MRA has been available since the beginning of MR imaging and is routinely used for intracranial imaging. It has also been validated for use in coronary, thoracic, renal and peripheral vascular disease [30]. In a recent review, Provenzale et al. [31] found similar diagnostic quality in MRI combined with MRA compared to CTA for carotid and vertebral dissection without clear superiority of either method. TOF MRA has also been compared to computed tomography angiography (CTA) and digital subtraction angiography (DSA) in following treated cerebral aneurysms, and has high sensitivity in detecting residual flow within the aneurysm [32].

**Figure 7:**
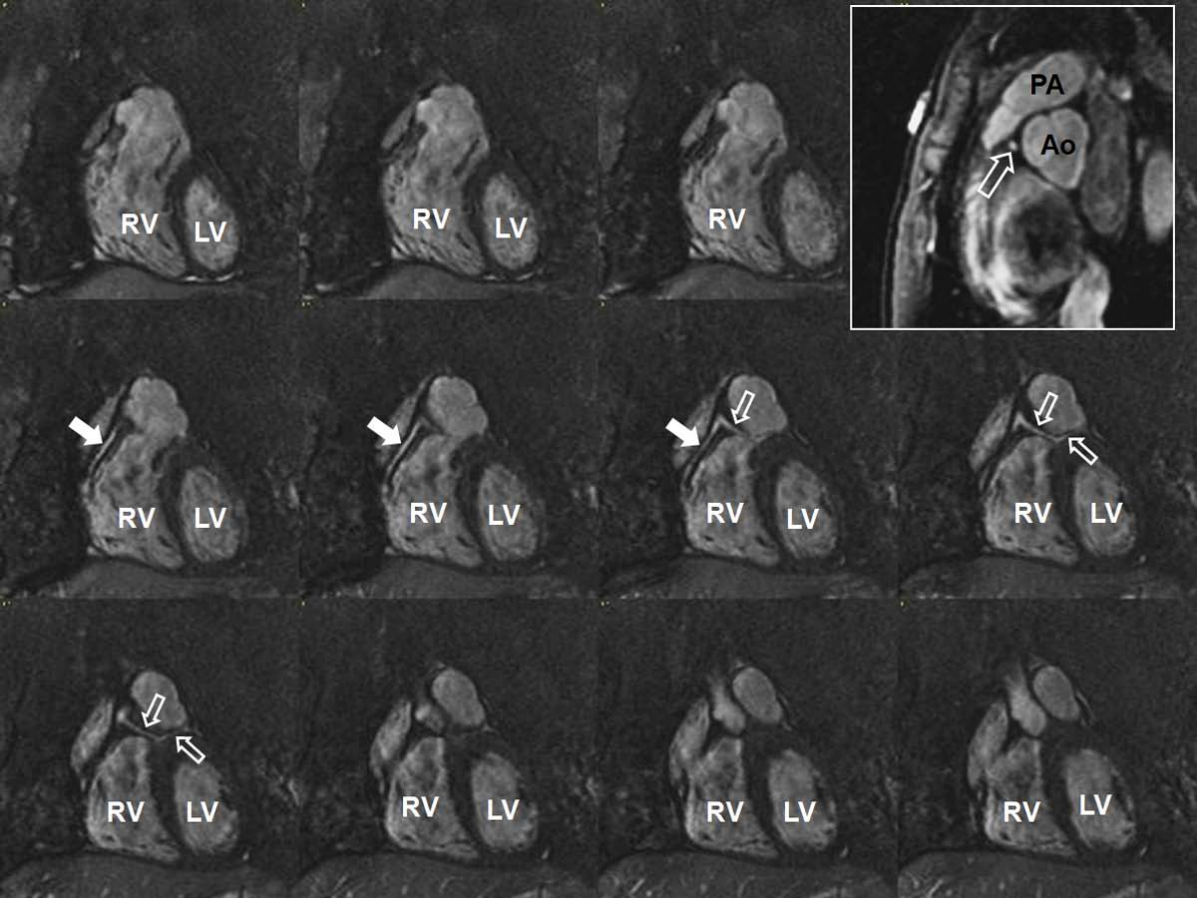
**Coronary MRA with 3D steady-state free precession**. The left main coronary artery (open arrow) arises from the right coronary artery (closed arrow) and courses between the pulmonary artery and aorta (inset). LV = left ventricle; RV = right ventricle; PA = pulmonary artery; Ao = aorta.

Coronary MRA has been validated primarily at 1.5T [33-37], but its clinical use has been limited by limitations in visualizing distal segment and small branch disease [38]. and the widespread introduction of coronary CTA. However, coronary MRA still has a role in the evaluation of anomalous coronary artery origins (Figure 7), particularly in pediatric patients. In addition, coronary MRA may have a role in evaluating patients with significant stenosis in coronary artery segments with moderate to severe calcification, due to increased artifact and difficulty visualizing stenosis with CTA in patients with high calcium scores [39]. Additionally, at 3.0T the improved SNR can increase visibility of distal coronary artery segments and shorter imaging time can improve image sharpness [40]. Due to the increased artifacts with SSFP sequences at 3.0T, contrast-enhanced coronary MRA methods have been revisited with promising initial results [41-45].

#### Time-of-Flight MRA

Time-of-flight (TOF) is the most commonly used NCE MRA technique, especially for peripheral and intracranial applications. TOF relies on the suppression of the background signal by rapid slice-selective radiofrequency excitation pulses that saturate the signal from stationary tissue, resulting in suppressed background signal [30,46]. Because the venous signal could potentially obscure the visualization of the adjacent arteries, the venous flow is usually selectively suppressed by applying a saturation band on the venous side of the imaging slice to null the signal as it enters the slice being imaged. This same principle can be applied to the diaphragm during respiration and the heart during the cardiac cycle. In tissue planes with high flow velocity, the incoming blood will be free of the excitation pulse that saturates the background tissues resulting in strong signal intensity. Slow blood flow or stasis, retrograde filling, tortuous vessels, or vessels in the same plane as the image slice result in saturation of the blood flow in the image volume and poor vessel visualization.

**Figure 8:**
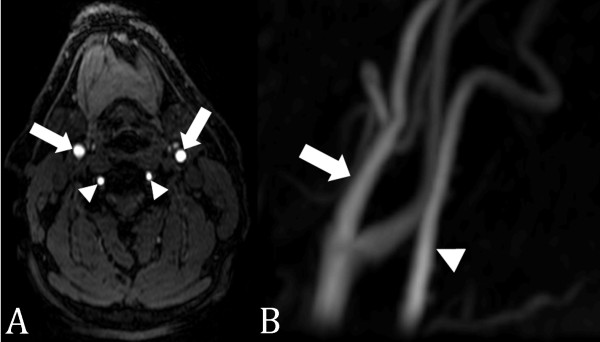
**2D time-of-flight MRA of the carotid arteries**. (A) Axial source image with excellent vascular signal in the carotid (arrows) and vertebral (arrowheads) arteries. (B) Maximum intensity projection image of the left carotid (arrows) and vertebral (arrowheads) arteries.

**Figure 9:**
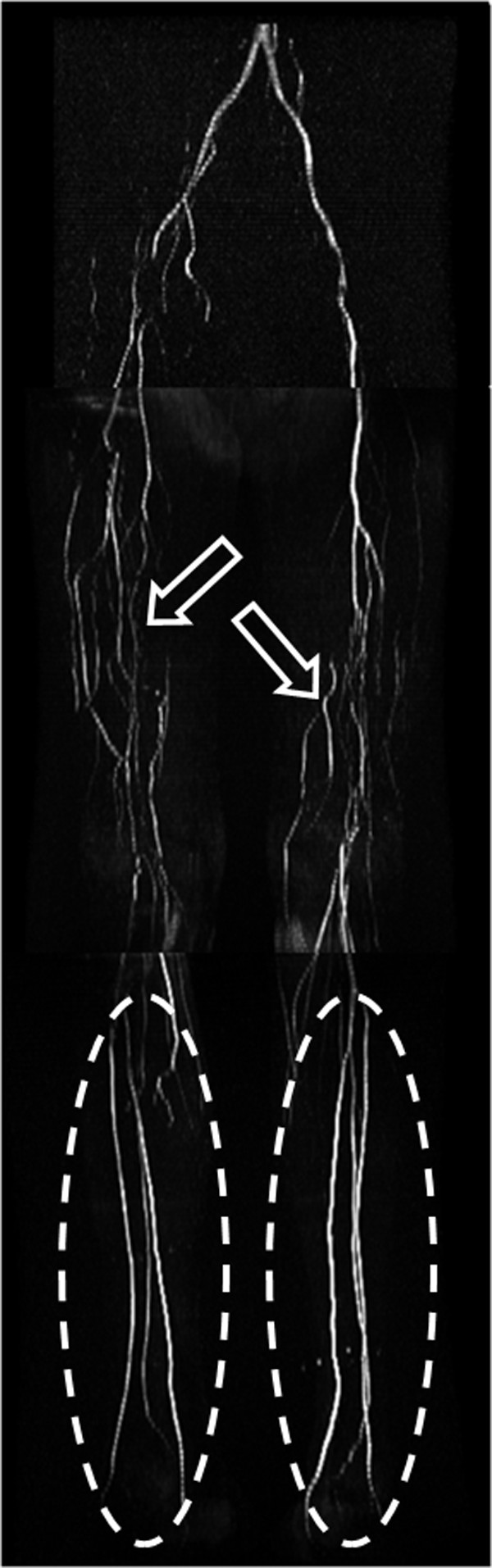
**2D time-of-flight MRA runoff**. 2D time-of-flight MRA of the pelvis, thighs, and calves in a patient with bilateral lower extremity claudication due to occlusion of the superficial femoral arteries bilaterally. Flow to the runoff vessels in the calves (ellipses) is through collateral arteries (open arrows) in the thighs arising from the profunda femoris arteries.

TOF acquisitions can be performed using 2D or 3D sampling, with 3D TOF being most commonly used for intracranial vasculature due to the tortuous nature of the arterial tree, tendency for flow within the imaging plane, and need for high spatial resolution [46]. 2D TOF angiography is used more often clinically in the evaluation of the carotid arteries (Figure 8) and peripheral vasculature (Figure 9), which is oriented orthogonal to the imaging plane [47]. While the saturation of protons within the in-plane vessels is the greatest limitation of TOF, it can be overcome by the use of progressively increasing flip angles through the slab to compensate for the saturation of blood flowing into the slab [48], multiple overlapping thin slab acquisition (MOTSA), which acquires the image volume as multiple thin 3D slabs and has less signal saturation than in a single-volume 3D acquisition [49].

ECG-gating has been successfully applied to CE-MRA techniques in the thoracic aorta, where cardiac motion can result in blurring of the vessel wall in the ascending portion of the aorta [50]. For imaging the peripheral arteries, where blood flow depends on the phase of the cardiac cycle, systolic gating can be used to time the image acquisition during peak blood flow [30]. Lanzman et al. [51]recently describe the use of a promising novel ECG-gated 3D NCE-MRA technique in patients with peripheral artery disease, showing adequate image quality and disclosure of significant arterial stenoses in the lower extremities without the need for exogenous contrast media.

#### Steady-State Free Precession MRA

**Figure 10:**
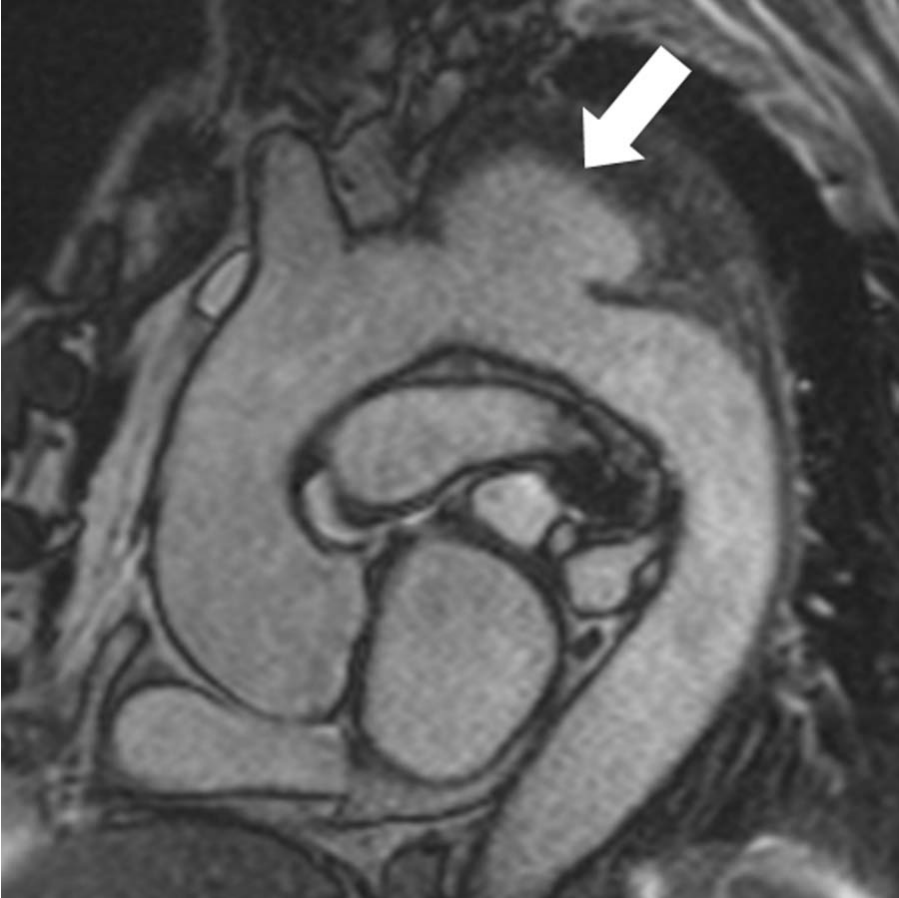
**SSFP thoracic MRA**. Non-contrast-enhanced SSFP MRA in a patient with a saccular aortic arch aneurysm (arrow).

Balanced steady-state free precession (SSFP) techniques are popular for NCE MRA because image contrast is determined by T2/T1 ratios, which leads to inherently bright blood images with little dependence upon blood inflow [30]. Both arteries and veins have bright signal with SSFP MRA, which makes this technique well suited for thoracic MRA applications (Figure 10) where the vessels are larger and where evaluation of both arterial and venous structures is important (i.e. in congenital heart disease). In clinical scenarios where venous signal may interfere with the interpretation of the MRA (i.e. renal MRA), venous inflow suppression techniques can be applied to SSFP MRA techniques to obtain purely arterial MRA images.

In a retrospective analysis by François et al. [52] of 23 patients who underwent both CE-MRA and 3D SSFP of the thoracic aorta, measurement of the aortic diameter was essentially equal between the two methods with notably superior visualization of the aortic root using 3D SSFP. A separate study compared CE-MRA to 3D SSFP for the evaluation of pulmonary veins (PV) prior to radiofrequency ablation surgery, and the 3D SSFP images demonstrated accurate PV diameter measurements with superior SNR and CNR [53]. A study by Krishnam et al. [54] demonstrated that free-breathing ECG-gated SSFP MRA of the thoracic aorta had equal diagnostic sensitivity and specificity compared to CE-MRA in 50 patients with suspected thoracic aorta disease. Independent qualitative and quantitative image analysis showed both techniques providing excellent visibility grades of all aortic segments. SSFP MRA demonstrated better visibility of the aortic root and had higher SNR and CNR values for all segments, while allowing the patient to breathe freely during imaging.

3D SSFP MRA has also been applied to the evaluation of the renal arteries. Maki, et al. [55]compared 3D SSFP MRA to CE-MRA at 1.5T in 40 patients and showed that 3D SSFP MRA had a sensitivity of 100% and specificity of 84%. Similarly, Wyttenbach, et al. [56] evaluated 53 patients suspected of renal artery stenosis with 3D SSFP and CE-MRA at 1.5T, with 3D SSFP MRA having a sensitivity and specificty of 100% and 84%, respectively. A study by Lanzman et al. [57] compared the image quality and visibility of renal arteries at 1.5T and 3.0T and demonstrated a significant gain in SNR and CNR at 3.0T of 13-16% and 16-23% respectively, with the greatest improvement of mean image quality at the segmental artery branches. The gain, while significant, is less than expected by the theoretically doubling of SNR anticipated at 3.0T due to SSFP relying on contrast from T2/T1 ratio.

**Figure 11:**
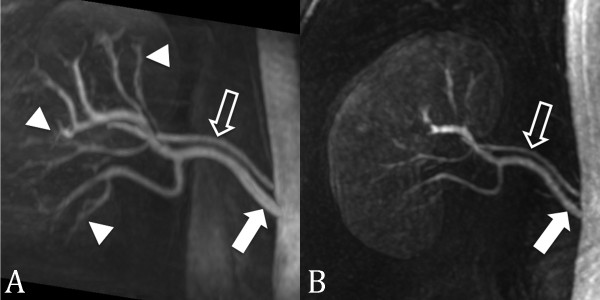
**SSFP renal MRA**. (A) Non-contrast-enhanced, inflow prepared, inversion recovery SSFP MRA and (B) contrast-enhanced MRA in a patient with two right renal arteries (closed arrow = main renal artery; open arrow = accessory renal artery). Interestingly, the segmental renal artery branches (arrowheads) are better seen with SSFP MRA than with contrast enhanced MRA.

**Figure 12:**
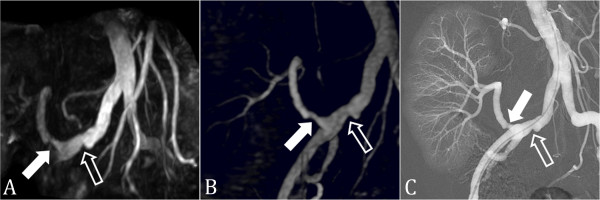
**SSFP renal transplant MRA**. (A) Non-contrast-enhanced, inflow prepared, inversion recovery SSFP MRA, (B) contrast-enhanced MRA, and (C) digital subtraction angiography in a patient with renal transplant artery stenosis (closed arrow). A stenosis is also present in the common iliac artery (open arrow).

Arterial spin labeling (ASL) is a technique that can be combined with SSFP to enhance image quality through improved background tissue suppression. Protons upstream of the imaging field are "tagged" with an inversion pulse to provide contrast. Background tissue can be suppressed by subtracting the untagged image from the tagged blood image in two acquisitions [58] or by applying a spatially nonselective tag pulse of the entire imaging field in addition to the tag pulse applied to the arteries of interest in a single acquisition [30]. ASL with SSFP provides bright-blood, venous-free images with high SNR especially suited for carotid and renal artery imaging (Figure 11) due to decreased sensitivity to flow artifacts [30]. The complex vasculature of the aorta relative to the renal arteries is well visualized in this technique, and initial clinical experience has shown comparable results to CE-MRA in both healthy volunteers and patients with renal artery stenosis (Figure 12) [59,60]. Using this type of sequence in 67 patients suspectec of renal artery stenosis, Glockner et al. [61] found that SSFP provided diagnostic images in most cases, but having a higher incidence of false positive and negative results compared to CE-MRA.

ASL is limited by relying on arterial velocity to replace blood in the imaging plane with tagged blood. In peripheral arteries with slower flow, the inflow of tagged blood can approach the T1 of the surrounding tissues, thus eliminating the tagging effect. This can be partially overcome by the multiple, thinner-slab acquisitions, but at the expense of longer imaging times.

#### Phase-Contrast MRA

**Figure 13:**
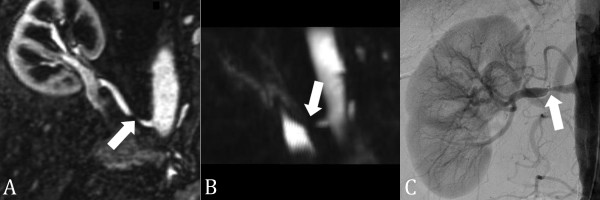
**3D phase contrast MRA**. (A) Contrast-enhanced MRA, (B) 3D phase contrast (PC) MRA, and (C) digital subtraction angiography in a patient with right renal artery stenosis (arrow). The signal void on the 3D PC MRA indicates that the stenosis is hemodynamically significant. The pressure gradient across the stenosis at catheter angiography was 18 mmHg.

Phase-Contrast (PC) MRA generates an image by applying a bipolar velocity-encoding gradient during the pulse sequence twice in opposing directions, which results in a net phase change of zero in stationary tissues while applying a phase change in moving blood, producing a signal. Signal strength is proportional to the velocity of moving blood, and the strength of the bipolar flow encoding gradient, which is prescribed by setting the Velocity Encoding (Venc) value. The Venc describes the maximum velocity that can be accurately encoded without aliasing, similar to Doppler velocity measurement. Thus, phase-contrast MRA provides anatomic images of vessels, in addition to hemodynamic data, about flow, unlike TOF and CE-MRA techniques. The intravascular signal loss on 3D PC MRA at and distal to a hemodynamically significant stenosis (Figure 13) is due to intravoxel phase dispersion related to turbulent flow, and can be used to estimate the hemodynamic significance of stenoses [62]. PC MRA can be used to identify the direction and velocity of flow, and has better background suppression compared to TOF. Its use is limited by longer image acquisition times and higher sensitivity to changes in velocity and magnitude of blood flow during the cardiac cycle [63]. At 3.0T, although there is not increased accuracy of flow measurements compared to 1.5T, there is greater signal and less noise measured for a given VENC. This allows VENC to be increased, reducing aliasing artifacts in regions of higher flow without increasing overall image noise to unacceptable levels [64].

**Figure 14:**
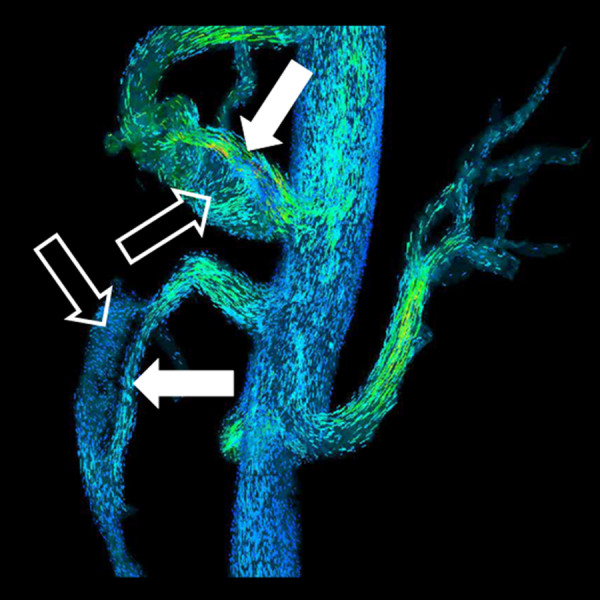
**4D flow MRA**. Particle traces from 4D flow MRA (PC VIPR) in same patient in Figure 1. Laminar flow is present in the true lumen (closed arrow) and helical flow is present in the false lumen (open arrow).

Traditionally, PC MRA was performed with three-directional velocity encoding without any temporal information to obtain a "complex-difference" MR angiogram. With this approach, each acquisition was repeated three times with a different velocity-compensation direction and once without flow compensation. Because four acquisitions are needed for 3D PC MRA, the scan times are long and the imaging volume is limited. Parallel imaging techniques [65] and 3D radial undersampling [66], or Vastly undersampled Isotropic Projection Reconstruction (VIPR), have been used to reduce scan time without compromising coverage or resolution. In addition, these image acquisition acceleration techniques have enabled the acquisition of temporal information in addition to the standard 3D PC MRA acquisition, resulting in four-dimensional (4D = three-dimensional spatial encoding, three-directional velocity encoding, and time) PC MRA for a variety of vascular applications. While these newer 4D PC MRA sequences can be used for NCE MRA, the future direction of these techniques lies in the additional hemodynamic information provided. In contrast to conventional two-dimensional (2D) PC MRA, where the vessel of interest must be known prior to scanning and the image plane must be prescribed at the scanner during the examination, 4D PC MRA techniques permit the *post priori *evaluation of flow velocities of any vessel within the imaging volume from the same acquisition. In addition, the 4D PC MRA techniques can be used to qualitatively evaluate the complex flow patterns within the cardiovascular system (Figure 14) [65,67-69] and calculate various hemodynamic parameters non-invasively, including pressure gradients [70,71,61], wall shear stress, and oscillatory stress index [68,72]. Implementation of these techniques into clinical routine is currently limited by our ability to process and interpret the large amount of data generated by these sequences.

## Conclusions

In summary, recent improvements in MRI hardware and software have lead to dramatic changes in the techniques used for MRA. The greater use of 3.0T scanners for MRA combined with improved parallel imaging methods have brought about a paradigm shift in CE-MRA toward a "less is more" approach. Further reductions in intravenous contrast administration have been made possible with the availability of novel intravascular contrast agents. The other recent major development in MRA has been the renewed use of NCE-MRA methods. Although NCE-MRA methods still require longer scan times than CE-MRA methods, they do offer several advantages over CE-MRA, including reduced risk to patients and lower costs. Interestingly, phase-contrast NCE-MRA methods offer the potential to provide additional hemodynamic information that currently is obtained using invasive methods.

## Competing interests

The authors declare that they have no competing interests.

## Authors' contributions

All authors participated in literature review, manuscript preparation and final approval of submitted manuscript.
